# Comparison of two extended depth of focus intraocular lenses with a monofocal lens: a multi-centre randomised trial

**DOI:** 10.1007/s00417-020-04868-5

**Published:** 2020-09-11

**Authors:** Thomas Reinhard, Philip Maier, Daniel Böhringer, Eckart Bertelmann, Tobias Brockmann, Laszlo Kiraly, David Salom, Matteo Piovella, Stephane Colonval, Javier Mendicute

**Affiliations:** 1 Klinik für Augenheilkunde, Universitätsklinikum Freiburg, Killianstrasse 5, 79106 Freiburg, Germany; 2 Medizinische Fakultät, Albert-Ludwigs-Universität Freiburg, Freiburg, Germany; 3 Charité – Universitätsmedizin, Berlin, Germany; 4 Universitätsmedizin, Rostock, Germany; 5 Augenlaserzentrum, Leipzig, Germany; 6 Hospital de Manises, Valencia, Spain; 7 Centro Microchirurgia Ambulatoriale, Monza, Italy; 8 Centre Hospitalier Jolimont Lobbes, Binche, Belgium; 9 Hospital Universitario, Donostia, Spain

**Keywords:** Cataract, presbyopia, Depth of focus, Intermediate visual acuity, Near visual acuity, EDOF, Defocus

## Abstract

**Purpose:**

The AT LARA 829MP is a next-generation extended depth of focus (EDOF) intraocular lens (IOL) providing continuous vision over a range of distances. The aim of this prospective multi-centre randomised trial was to compare two EDOF IOLs and one monofocal IOL.

**Methods:**

Cataract patients between 50 and 80 years were randomised for bilateral implantation with either the AT LARA 829MP (EDOF), the TECNIS Symfony (EDOF) or the CT ASPHINA 409MP (monofocal). Follow-up was at 1 to 2 weeks, 1 month and 4 to 6 months.

**Results:**

A total of 211 patients were randomised and included in the final analysis. Monocular depth of focus was significantly better for AT LARA 829MP eyes compared with that for TECNIS Symfony at all thresholds (*p* = 0.024, 0.001 and 0.006, for 0.1, 0.2 and 0.3 logMAR respectively) with no significant difference for binocular depth of focus. LARA eyes had significantly better monocular depth of focus at all levels compared with ASPHINA eyes (all *p* < 0.0001), while there was no significant difference between Symfony and ASPHINA eyes at 0.1 logMAR and 0.2 logMAR. Both EDOF IOLs were significantly better than the monofocal ASPHINA at all levels for binocular depth of focus (LARA: all *p* < 0.0001; Symfony: all *p* = 0.002). Distance visual acuity was similar for all IOLs at 6 months; intermediate and near visual acuity were significantly better for the EDOF IOLs than for the monofocal (*p* < 0.0001). Refraction improved in all groups relative to baseline. Contrast sensitivity was higher with the CT ASPHINA 409MP but both EDOF lenses had a better spectacle independence rate. At 6 months, all IOLs were well centred with no cases of tilt. No general safety issues were raised for any of the groups.

**Conclusion:**

The two EDOF intraocular lenses investigated provided good visual outcomes with comparable visual acuity at all distances. The AT LARA 829MP provided the widest monocular depth of focus at 0.1 and 0.2 logMAR, with a clear superiority compared with the monofocal IOL. TECNIS Symfony was superior to the monofocal control at 0.3 logMAR. Spectacle independence and patient satisfaction were comparable.

**Trial registration:**

Trial registered on https://clinicaltrials.gov/ under the identification NCT03172351 (date of registration 1 June May 2017).

## Introduction

There is an increasing expectation by patients for spectacle-free vision following cataract surgery. Although excellent for distance vision, monofocal intraocular lenses (IOLs) generally cannot provide clear vision over a full range of distances including intermediate and near, while many patients demand improved vision for activities such as driving and use of devices such as computers and mobile phones. Options to correct for presbyopia do exist (multifocal and monovision, in particular) but have inherent problems. In multifocal lenses, the incoming light is split in such a way that light from different distances is focused at the same plane on the retina so that the viewer can visualise objects at more than one distance. In the case of a trifocal lens, the simultaneous images for distance, intermediate and near, are superimposed on the retina and neural processing is employed to filter and to create a sharp vision over a wide range of distances simultaneously [1]. Common drawbacks, however, are the occurrence of dysphotopsia such as glare and halos, and decreased contrast sensitivity compared with monofocal lenses [2]. With monovision, one eye is targeted for emmetropia and the other is targeted for near or intermediate distance. However, this principle is not tolerated by everyone and has to be tested prior to surgery. Furthermore, due to the presence of cataract, it is not always straightforward in practice to detect which eye is dominant or whether dominance is maintained for all distances.

For individuals who wish for a continuous range of functional vision, extended depth of focus (EDOF) IOLs have been developed in which incoming waves of light are focused in an extended longitudinal plane, instead of discrete points, to avoid overlapping of near and far images. There are currently few IOLs that can be classified as EDOF. Amongst them, the AT LARA 829MP (Carl Zeiss Meditec) received CE-marking in 2017 and the TECNIS Symfony (Johnson & Johnson, formerly Abbott Medical Optics) which was launched in 2014. The AT LARA 829MP features two additional focus planes at far-intermediate and intermediate distances enabling continuous visual acuity over a range of distances from far to intermediate-near. The TECNIS Symfony has a biconvex, wavefront-designed anterior aspheric surface, a posterior achromatic diffractive surface with echelettes to extend depth of focus [3–5]. The IOL is also optimised to correct chromatic and spherical aberration. Clinical studies for the TECNIS Symfony report better uncorrected vision in the intermediate and near range, as compared with monofocal IOLs [6–10]. No comparative studies have yet been published with the AT LARA 829MP.

The aim of this study was to compare the depth of focus and visual acuity of two EDOF IOLs—the AT LARA 829MP and the TECNIS Symfony IOL—and one monofocal IOL, the CT ASPHINA 409MP (Carl Zeiss Meditec).

## Methods

### Study design

This was a prospective multi-centre, randomised trial, with three arms comparing two EDOF IOLs with a monofocal IOL. The study was conducted in nine sites located in Germany, Belgium, Portugal, Spain, France and Italy.

Patients in each arm were randomised for bilateral implantation with the same IOL in each eye. Due to differences in the appearance of the IOLs, masking of the surgeons was not possible, but patients were masked to the identity of the implanted IOL and all visual performance parameters, including the depth of focus measurements, were assessed by masked examiners.

The trial was carried out in accordance with the ethical principles stated in the Helsinki Declaration and subsequent modifications regarding Good Clinical Practice. The trial (ClinicalTrials.gov, number NCT03172351) was approved by the relevant Competent Authority and Ethics Committee in each participating country and institution. Patients gave written informed consent before entering the study.

### Patients and IOLs

The study included patients aged between 50 and 80 years with clinically significant bilateral age-related cataract, and no other major ocular pathologies. The main exclusion criteria were as follow: any ocular disorders, other than cataract, that could potentially cause future acuity loss, any anterior segment pathologies that could significantly affect outcomes (e.g. chronic uveitis, iritis, corneal dystrophy), any type of corneal disorders, any eye infection, any degenerative visual disorders, pseudoexfoliations syndrome, keratoconus, diabetic retinopathy, uncontrolled glaucoma and or IOP > 24 mmHg, choroidal haemorrhage, aniridia, microphthalmia, amblyopia, previous intraocular and corneal surgery, expected post-operative astigmatism greater than 1 D. Three IOLs were under investigation: the AT LARA 829MP, the TECNIS Symfony and the CT ASPHINA 409MP (see Table 1 for details of the devices).

**Table 1:** Characteristics of the devices under study

	AT LARA 829MP	TECNIS Symfony	CT ASPHINA 409MP
Picture			
Type	Extended depth of focus (EDOF)	Extended depth of focus (EDOF)	Monofocal
Optic design	Biconvex aspheric Achromatic diffractive anterior surface	Wavefront-designed aspheric anterior surface Achromatic diffractive and echelette feature on posterior surface	Biconvex, aspheric, refractive
Material	Hydrophilic with hydrophobic surface	Hydrophobic	Hydrophilic with hydrophobic surface
Refractive index	1.47	1.47	1.47
Haptics	4-haptic	C-loop	4-haptic
Optic diameter	6.0 mm	6.0 mm	6.0 mm
Total diameter	11.0 mm	13.0 mm	11.0 mm
Dioptre range	− 10.0 D to + 32.0 D (0.5 D increments)	+ 5.0 D to + 34.0 D (0.5 D increments)	0.0 D to + 10.0 D (1.0 D increments) + 10.0 D to + 30.0 D (0.5 D increments) + 30.0 D to + 32.0 D (1.0 D increments)

Patients were randomised in the ratio 1:1:1 to bilateral implantation of one of the three study devices. Randomisation was stratified by centre and by dominant eye and was carried out via an Interactive Web Response System. The first eye to be randomised was denoted the ‘primary’ eye and the contralateral eye as ‘supportive’. Each site was allowed to randomise and treat a maximum of 36 patients. Following surgery, patients were followed up for 4 to 6 months.

### Surgical technique

Surgeons could use their own preferred technique, but this was similar across centres and the three IOL groups. Topical, loco-regional or general anaesthesia was used followed by continuous curvilinear capsulorhexis. In the majority of cases, the IOL was inserted into the capsular bag using the recommended injector (BLUEMIXS 180, Carl Zeiss Meditec for the AT LARA 829MP and CT ASPHINA 409MP and UNFOLDER, Platinum 1 series, J&J for TECNIS Symfony).

All surgeries were performed using standard self-sealing clear corneal incision, capsulorhexis and conventional phacoemulsification. The recommended incision size was ≤ 2.4 mm for the TECNIS Symfony and ≤ 2.2 mm for AT LARA 829MP and CT ASPHINA 409MP. Emmetropia was targeted in all cases. At the end of the surgery, any residual ophthalmic viscoelastic device was thoroughly removed from the posterior chamber by irrigation, and side ports and main incision were sealed by hydration. Post-operative treatment and medication were given according to the routine procedure in each centre.

### Assessments

Before surgery, all patients underwent comprehensive evaluation including full medical history, slit lamp and dilated fundus examination, subjective refraction, determination of the dominant eye, monocular and binocular uncorrected (UDVA) and corrected (CDVA) visual acuity, biometry (IOLMaster, Carl Zeiss Meditec), photopic pupil size and intraocular pressure (IOP). Follow-up examinations at 1–2 weeks, 1 month and 4–6 months measured visual acuity, subjective refraction, IOP, biometry parameters and IOL stability (slit lamp examination). Defocus curves were measured at 1 month. The 1 month and final examinations also measured pupil size, spectacle dependence and quality of life and the final examination also measured contrast sensitivity, PCO (posterior capsule opacification) and Nd:YAG rate.

Monocular and binocular uncorrected and distance corrected visual acuity was measured using the Freiburg Visual Acuity and Contrast Test (FrACT) computerised charts (available at http://www.michaelbach.de/fract.html) at 100% contrast at the following distances: far 400 ± 12 cm; intermediate 67 ± 2 cm; near 40 ± 1 cm. The luminance of the charts was standardised at 200 cd/m^2^. All visual acuity measurements are in logMAR. The test chart for refraction was the ETDRS chart at a distance of 4 m (± 12 cm).

The same measurement conditions and technique were used for measuring the monocular and binocular defocus curves. The patient’s vision was best-corrected for the testing distance (4 m) and visual acuity was measured with additions of defocus lenses from + 1.50 D to − 4.00 D in a randomised order. Defocus steps were 0.50 D. In this study, three visual acuity thresholds were used: 0.1, 0.2 and 0.3 logMAR. Depth of focus was calculated according to the ANSI Z80.35-2018 recommendations. The dioptric range between 0 defocus (or best distance vision) and the point corresponding to the largest negative defocus that crosses the visual acuity threshold was calculated for each patient individually. Then, the mean of all the individual measurements was calculated to allow group comparison. This evaluation is later referred in this manuscript as the one-sided depth of focus. In addition, the defocus curves were calculated from the mean visual acuities obtained at each defocus step. The defocus curves were two-sided, ranging from − 4.00 D to + 1.50 D.

Contrast sensitivity was measured monocularly at 1.5, 3, 6, 12 and 18 cpd spatial frequencies, under mesopic (3 cd/cm^2^), mesopic with glare and photopic (85 cd/cm^2^) conditions, using the Functional Vision Analyzer Optec 6500 Vision Tester (Stereo Optical). After adaptation to the dark, mesopic testing was carried out first, followed by photopic. Each test was carried out two or three times with the average of the readings used for the analysis. For each spatial frequency, the number of subjects who could not see any contrast was recorded.

Monocular measurements of defocus curve and contrast sensitivity were performed on the primary eye, selected through a randomisation process aiming for an equal number of dominant and non-dominant eyes.

### Objectives

The primary endpoint of the study was to evaluate the monocular depth of focus of the two EDOF lenses versus the monofocal CT ASPHINA 409MP IOL. The two key secondary objectives were to demonstrate the superiority of both EDOF IOLs over the monofocal IOL regarding depth of focus at the same level. Other secondary objectives included assessment of binocular depth of focus at 1 month, uncorrected and corrected distance, intermediate and near visual acuity up to 6 months, photopic and mesopic contrast sensitivity, subjective refraction, visual disturbances, IOL stability, PCO, Nd:YAG rate and general safety.

### Statistical methods

Based on an expected difference between the groups of at least 0.23 D at 0.1 logMAR, a power of 90% and a significance level of 0.05, the minimal recommended number of patients per treatment group was 58. Taking into account a possible loss to follow-up of 10%, and in order to have equal numbers in each arm at each site, 72 patients per arm were required.

The safety population was defined as all patients who have received an investigational device in at least one of their eyes. The full-analysis set was defined as all patients who had at least nine out of the 12 measurements for the monocular defocus curve at the 1-month visit.

The primary endpoint was analysed using the ANCOVA model with fixed terms for treatment, centre, dominant eye, first operated eye, time between surgery and the 1-month measurement. Statistical analysis of the primary and key secondary endpoints was performed at the 5% global significance level, using two-sided tests in a hierarchical test procedure. The analysis was carried out with SAS software version 9.3 or higher.

## Results

Two hundred and thirty-three patients were randomised: the safety population consisted of 215 patients bilaterally implanted with one of the three IOLs between June 2017 and December 2018 and the full-analysis set consisted of 211 patients. The three groups were similar in age, baseline visual acuity and refraction (Table 2). There were no serious complications during surgery.

**Table 2:** Baseline characteristics and refraction

	AT LARA 829MP	TECNIS Symfony	CT ASPHINA 409MP
Baseline characteristics			
Safety population, *n*	78	69	68
Age, mean ± SD, years	68.9 ± 7.36	69.4 ± 7.22	71.0 ± 6.52
Gender, M:F	48.7: 51.3	37.7: 62.3	36.8: 63.2
Target SE, mean ± SD, D	− 0.06 ± 0.17	− 0.06 ± 0.16	− 0.07 ± 0.16
IOL power, mean ± SD, D	21.23 ± 1.81	21.83 ± 2.06	21.02 ± 2.04
Pupil size (photopic), mean ± SD, mm	3.52 ± 1.04	3.27 ± 0.77	0.34 ± 0.78
Primary eye, dominant/non-dominant	48.7%/51.3%	47.8%/52.2%	50%/50%
Pre-operative biometry			
Full-analysis set, *n*	78	67	66
AL, mean ± SD, mm	23.26 ± 0.76	23.40 ± 0.71	23.35 ± 0.76
ACD, mean ± SD, mm	3.07 ± 0.36	3.17 ± 0.29	3.17 ± 0.39
Pre-operative visual acuity (logMAR) and refraction			
Monocular UDVA, mean ± SD	0.65 ± 0.33	0.65 ± 0.33	0.63 ± 0.31
Binocular UDVA, mean ± SD	0.51 ± 0.31	0.54 ± 0.31	0.50 ± 0.28
Monocular CDVA, mean ± SD	0.35 ± 0.30	0.37 ± 0.28	0.33 ± 0.29
Binocular CDVA, mean ± SD	0.24 ± 0.29	0.28 ± 0.26	0.25 ± 0.26
Cylinder, mean ± SD, D	− 0.66 ± 0.54	− 0.47 ± 0.46	− 0.60 ± 0.52
Sphere, mean ± SD, D	0.75 ± 2.07	0.44 ± 2.21	1.08 ± 1.90
SE, mean ± SD, D	0.16 ± 2.08	− 0.49 ± 2.21	0.51 ± 1.86
Post-operative refraction (primary eye, 4–6 months)			
Cylinder, mean ± SD, D	− 0.41 ± 0.48	− 0.41 ± 0.50	− 0.39 ± 0.41
Sphere, mean ± SD, D	0.18 ± 0.39	0.07 ± 0.37	0.14 ± 0.54
SE, mean ± SD, D	− 0.28 ± 0.34	− 0.39 ± 0.33	− 0.31 ± 0.47

*IOL*, intraocular lens; *AL*, axial length; *ACD*, anterior chamber depth; *UDVA*, uncorrected distance visual acuity; *CDVA*, corrected distance visual acuity; *SE*, spherical equivalent; *SD*, standard deviation; *D*, dioptre

### Depth of focus

Table 3 summarises the mean one-sided depth of focus values for the three visual acuity thresholds 0.1, 0.2 and 0.3 logMAR.

**Table 3:** Depth of focus (DoF) measured at 1 month at selected logMAR visual acuity thresholds for the three groups

	Monocular			Binocular		
	AT LARA 829MP	TECNIS Symfony	CT ASPHINA 409MP	AT LARA 829MP	TECNIS Symfony	CT ASPHINA 409MP
	*n* = 78	*n* = 67	*n* = 66	*n* = 78	*n* = 67	*n* = 66
0.1 logMAR						
DoF (mean (95% CI)), D	0.87 (0.66; 1.08)	0.61 (0.38; 0.84)	0.46 (0.23; 0.69)	1.25 (1.04; 1.46)	1.06 (0.83; 1.29)	0.67 (0.44; 0.90)
*p* value (AT LARA-XY)	–	0.024	< 0.001	–	0.105	< 0.0001
*p* value (Symfony-XY)	–	–	0.218	–	–	0.002
0.2 logMAR						
DoF (mean (95% CI)), D	1.36 (1.14; 1.57)	0.96 (0.73; 1.20)	0.74 (0.50; 0.97)	1.72 (1.50; 1.94)	1.51 (1.26; 1.75)	1.11 (0.87; 1.35)
*p* value (AT LARA-XY)	–	0.001	< 0.0001	–	0.093	< 0.0001
*p* value (Symfony-XY)	–	–	0.075	–	–	0.002
0.3 logMAR						
DoF (mean (95% CI)), D	1.75 (1.55; 1.96)	1.43 (1.21; 1.66)	1.18 (0.95; 1.41)	2.11 (1.88; 2.33)	1.90 (1.65; 2.15)	1.49 (1.24; 1.74)
*p* value (AT LARA-XY)	–	0.006	< 0.0001	–	0.106	< 0.0001
*p* value (Symfony-XY)	–	–	0.037	–	–	0.002

*DoF*, depth of focus; *CI*, confidence interval; *D*, dioptre

Monocular depth of focus was found to be significantly larger in the AT LARA 829MP group compared with that in the TECNIS Symfony group and the CT ASPHINA 409MP for all three visual acuity threshold values. At 0.1 and 0.2 logMAR, there were no statistically significant differences between the TECNIS Symfony and the CT ASPHINA 409MP groups; however, a statistically significant difference was found at 0.3 logMAR (*p* = 0.037) in favour of the TECNIS Symfony.

With regard to binocular depth of focus, there was no statistically significant difference between the two EDOF IOLs at any visual acuity level (*p* ≥ 0.093), but both were statistically superior to the CT ASPHINA 409MP group (*p* ≤ 0.002).

**Fig. 1:**
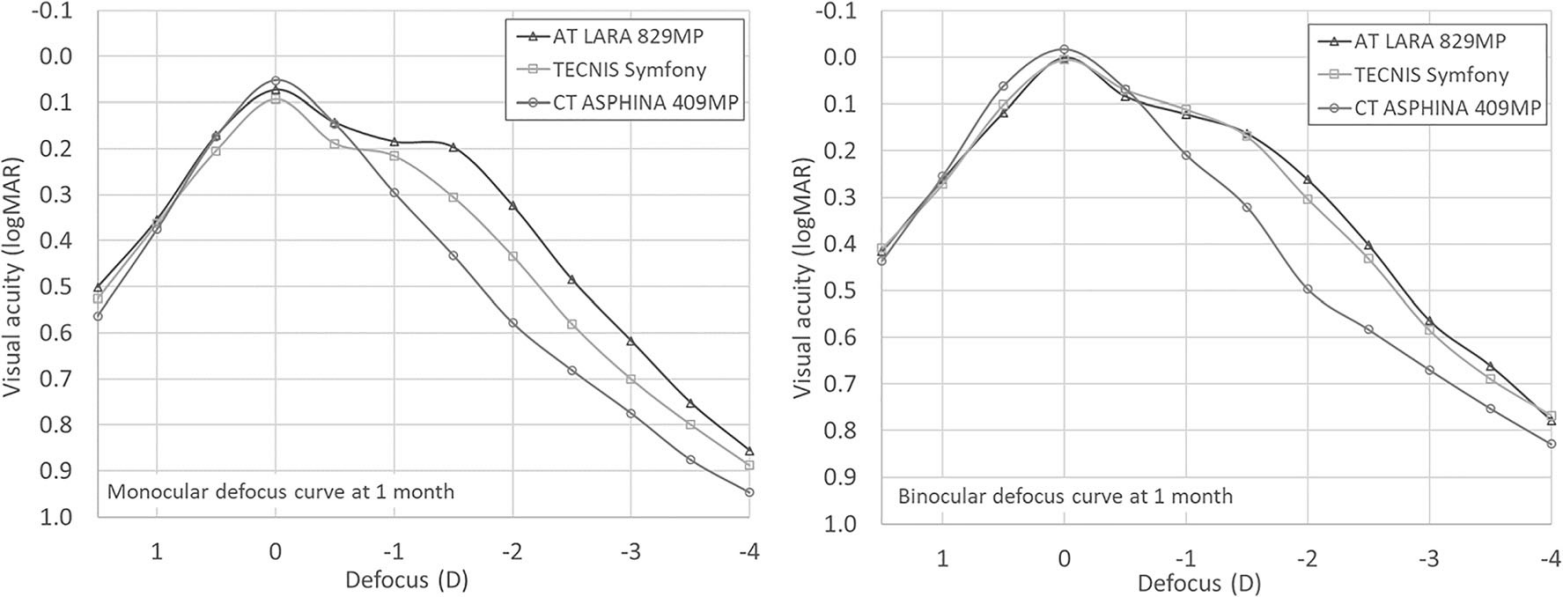
Mean monocular and binocular defocus curves measured 1 month post-operatively for the AT LARA 829MP, the TECNIS Symfony and the CT ASPHINA 409MP IOLs

The mean monocular defocus curves at 1 month (Fig. 1) show that the AT LARA 829MP maintained visual acuity of 0.3 logMAR or better through the range from + 0.85 D to − 1.9 D. For the same visual acuity threshold, the range for the TECNIS Symfony was + 0.8 D to − 1.5 D, and for the CT ASPHINA 409MP monofocal IOL, it was + 0.8 D to − 1.0 D. Regarding the binocular defocus at the same visual acuity threshold, the range of the defocus curves was + 1.3 D to − 2.15 D for the AT LARA 829MP, + 1.1 D to − 2.0 D for the TECNIS Symfony and + 1.15 D to − 1.4 D for the CT ASPHINA 409MP.

### Visual acuity: monocular and binocular

The monocular (primary eye) and binocular visual acuities for distance, intermediate and near vision, at 1 week and 6 months are shown in Tables 4 and 5. There was no significant difference between any group at 6 months for distance visual acuity—corrected or uncorrected, monocular or binocular.

**Table 4:** Mean monocular (primary eye) visual acuity, in logMAR

	1 to 2 weeks			6 months		
	AT LARA 829MP	TECNIS Symfony	CT ASPHINA 409MP	AT LARA 829MP	TECNIS Symfony	CT ASPHINA 409MP
	*n* ≥ 70	*n* ≥ 59	*n* ≥ 56	*n* = 76	*n* = 66	*n* = 65
UDVA	0.21 ± 0.23	0.19 ± 0.31	0.21 ± 0.25	0.12 ± 0.18	0.12 ± 0.19	0.14 ± 0.22
CDVA	0.12 ± 0.21	0.16 ± 0.32	0.08 ± 0.20	0.04 ± 0.16	0.05 ± 0.16	0.02 ± 0.16
UIVA	0.23 ± 0.30	0.27 ± 0.19	0.43 ± 0.39	0.16 ± 0.17	0.20 ± 0.18	0.42 ± 0.21
DCIVA	0.21 ± 0.31	0.23 ± 0.18	0.44 ± 0.36	0.12 ± 0.18	0.18 ± 0.17	0.37 ± 0.18
UNVA	0.34 ± 0.33	0.46 ± 0.20	0.66 ± 0.39	0.34 ± 0.22	0.39 ± 0.20	0.63 ± 0.19
DCNVA	0.37 ± 0.32	0.46 ± 0.20	0.68 ± 0.38	0.32 ± 0.19	0.39 ± 0.21	0.58 ± 0.24

*UDVA*, uncorrected distance visual acuity; *CDVA*, corrected distance visual acuity; *UIVA*, uncorrected intermediate visual acuity; *DCIVA*, distance corrected intermediate visual acuity; *UNVA*, uncorrected near visual acuity; *DCNVA*, distance corrected near visual acuity

**Table 5:** Mean binocular (primary eye) visual acuity, in logMAR

	1 week			4 to 6 months		
	AT LARA 829MP	TECNIS Symfony	CT ASPHINA 409MP	AT LARA 829MP	TECNIS Symfony	CT ASPHINA 409MP
	*n* ≥ 70	*n* ≥ 59	*n* ≥ 56	*n* = 76	*n* = 66	*n* = 65
UDVA	0.09 ± 0.26	0.07 ± 0.15	0.10 ± 0.26	0.02 ± 0.17	0.02 ± 0.16	0.01 ± 0.16
DCVA	0.02 ± 0.20	0.04 ± 0.22	−0.02 ± 0.23	−0.03 ± 0.14	−0.03 ± 0.13	−0.06 ± 0.15
UIVA	0.11 ± 0.29	0.17 ± 0.16	0.29 ± 0.38	0.07 ± 0.15	0.11 ± 0.14	0.30 ± 0.17
DCIVA	0.12 ± 0.29	0.13 ± 0.17	0.34 ± 0.38	0.05 ± 0.15	0.08 ± 0.14	0.29 ± 0.15
UNVA	0.18 ± 0.20	0.36 ± 0.16	0.53 ± 0.41	0.23 ± 0.23	0.30 ± 0.17	0.53 ± 0.18
DCNVA	0.28 ± 0.32	0.35 ± 0.17	0.52 ± 0.41	0.22 ± 0.18	0.30 ± 0.18	0.50 ± 0.23

*UDVA*, uncorrected distance visual acuity; *CDVA*, corrected distance visual acuity; *UIVA*, uncorrected intermediate visual acuity; *DCIVA*, distance corrected intermediate visual acuity; *UNVA*, uncorrected near visual acuity; *DCNVA*, distance corrected near visual acuity

Intermediate visual acuities were significantly better for the two EDOF IOLs than for the monofocal IOL (*p* < 0.0001) for corrected and uncorrected and monocular and binocular conditions, and comparable between the AT LARA 829MP and the TECNIS Symfony (*p* = 0.3413 monocular uncorrected, *p* = 0.0940 monocular distance corrected, *p* = 0.2354 binocular uncorrected and *p* = 0.3425 binocular distance corrected). Mean monocular DCIVA was better than 0.3 logMAR for 81.6% of the eyes in the AT LARA 829MP group, 77.3% in the TECNIS Symfony group and 38.5% in the CT ASPHINA 409MP group.

Near visual acuities were better for the two EDOF IOLs than for the monofocal IOL (*p* < 0.0001 for all measurements) and monocular and binocular distance corrected near visual acuities (DCNVA) were better for the AT LARA 829MP than for the TECNIS Symfony (*p* ≤ 0.01) while no difference was found between the two EDOF lenses for monocular and binocular UNVA (*p* ≥ 0.06).

Mean monocular DCNVA was better than 0.3 logMAR for 46.1% of the eyes in the AT LARA 829MP group, 27.3% in the TECNIS Symfony group and 15.4% in the CT ASPHINA 409MP group.

### Refraction

**Fig. 2:**
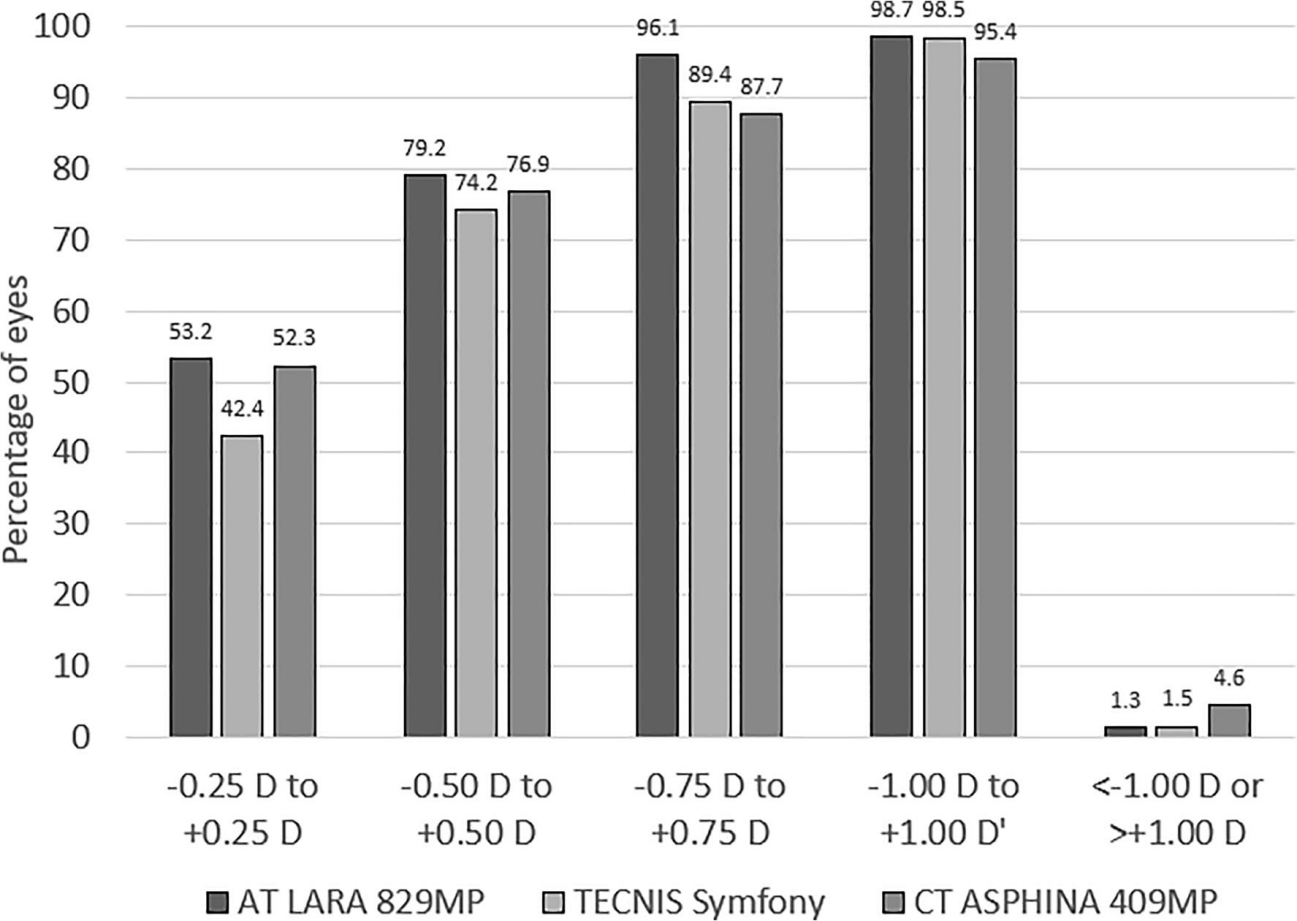
Post-operative spherical equivalent at 6 months for the AT LARA 829MP, the TECNIS Symfony and the CT ASPHINA 409MP

Refraction improved in all groups relative to baseline (see Table 2) and accuracy at 6 months was very similar across the three groups (Fig. 2).

The percentage of eyes that achieved ± 0.5 D of target spherical equivalent refraction was 79.2% for the AT LARA 829MP group, 72.7% for the TECNIS Symfony and 76.9% for the CT ASPHINA 409MP group. At ± 1.0 D of target, the values were 98.7% for the AT LARA 829MP group, 98.5% for the TECNIS Symfony and 95.4% for the CT ASPHINA 409MP group.

### Contrast sensitivity

**Fig. 3:**
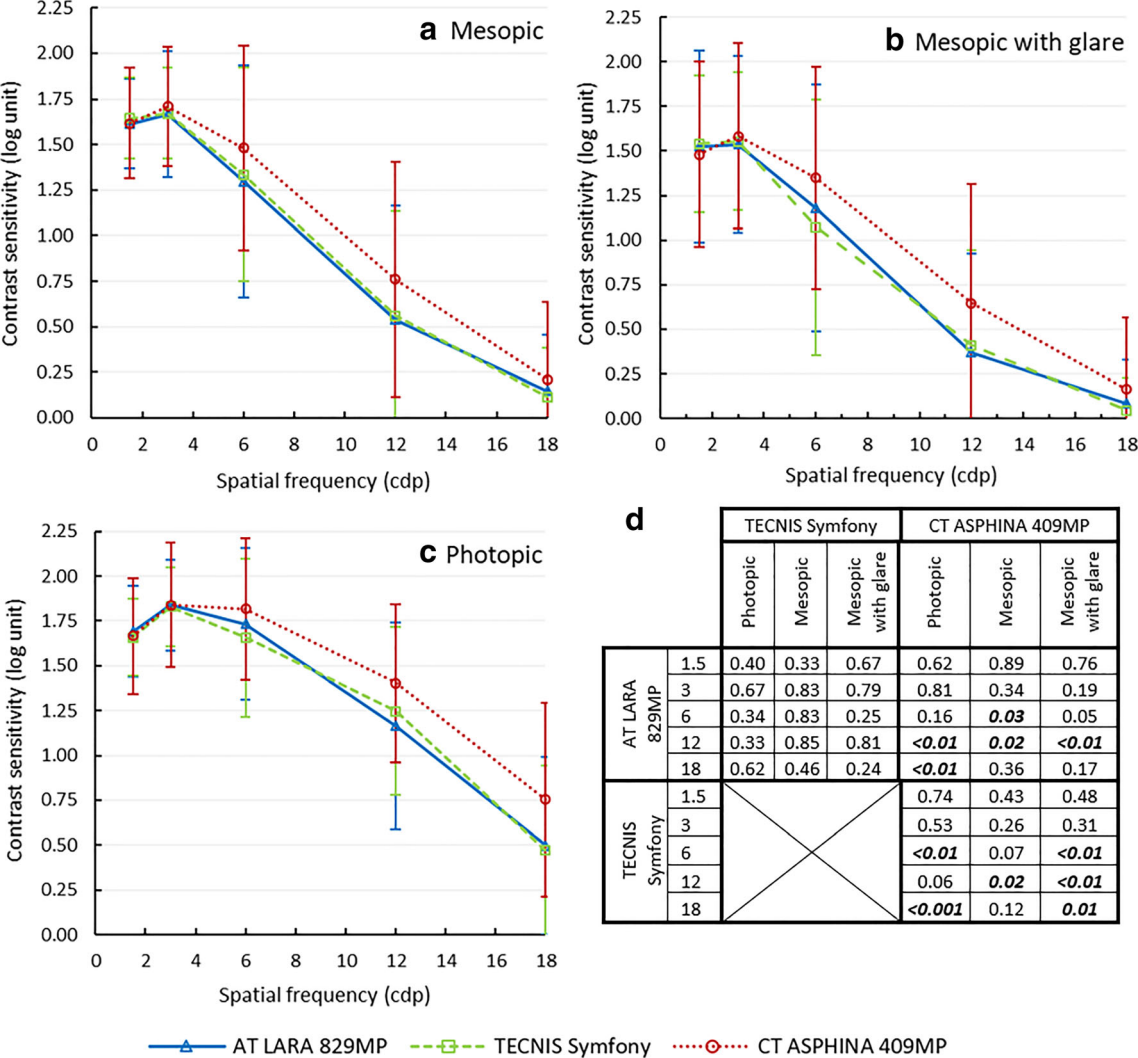
Contrast sensitivity curves with distance correction under mesopic (**a**), mesopic with glare (**b**) and photopic (**c**) conditions with *p* values from the ANCOVA group comparison (**d**) for the AT LARA 829MP, the TECNIS Symfony and the CT ASPHINA 409MP, 6 months post-operatively

Contrast sensitivity curves with distance correction measured at 6 months are shown in Fig. 3. In each group, contrast sensitivity was reduced under mesopic conditions and even further under mesopic with glare conditions compared with photopic conditions. Contrast sensitivity curve was higher with the monofocal IOL compared with that with the EDOF lenses but statistically significant differences were not only found for some spatial frequencies (see Fig. 3d). There were no significant differences between the two EDOF lenses at any spatial frequencies and under any of the three ambient lighting conditions (*p* ≥ 0.24).

### Patient questionnaire

**Fig. 4:**
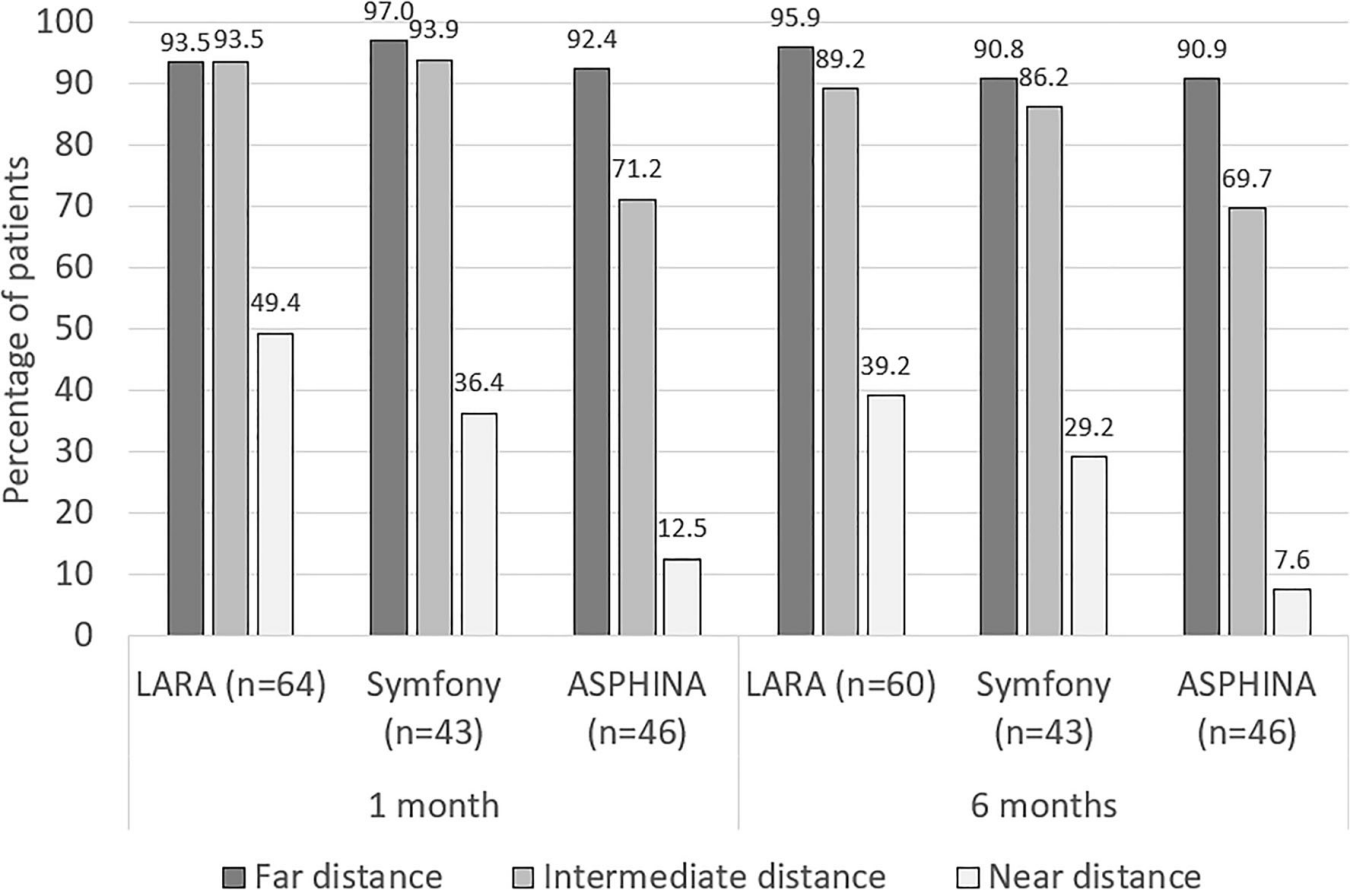
Spectacle independence rates for far, intermediate and near distances for the AT LARA 829MP, TECNIS Symfony and CT ASPHINA 409MP at 1 month and 6 months post-operatively

Summary of the patient questionnaire is given in Table 6 and the rates of spectacle independence for far, intermediate and near distances are shown in Fig. 4.

**Table 6:** Questionnaire outcomes at the 6 months follow-up for the 3 groups

	AT LARA 829MP (%)	TECNIS Symfony (%)	CT ASPHINA 409MP (%)
Because of your vision, how much difficulty do you have? (% of patients expressing no difficulty at all)			
- With your daily activities?	74.7	86.4	75.8
- Doing work or hobbies that require you to see well up close, such as cooking, fixing things around the house, sewing, using hand tools or working with a computer?	56.0	59.1	59.1
- Reading ordinary print in newspapers?	57.3	54.5	36.4
- Reading the small print in a telephone book, on a medicine bottle or on legal forms?	28.0	25.8	25.8
- Driving during the daytime in familiar places?	91.7	90.7	91.3
- Driving at night?	45.0	69.8	52.2
In the last 7 days, have you seen any? (% of patients observing no symptoms at all)			
- Double images?	96.0	100	98.4
- Glare?	64.9	66.2	62.1
- Halos?	46.7	59.1	87.5
- Starburst?	36.0	61.5	81.8

Regarding spectacle independence rates, there were no statistically significant differences between the two EDOF groups (*p* values of 0.5737, 0.5921 and 0.7098 at far, intermediate and near distances, respectively). At intermediate distance, a statistically significant difference was found between the AT LARA 829MP lens and the monofocal lens (*p* = 0.0144) but no difference was found between the TECNIS Symfony and the monofocal lens (*p* = 0.1123). At near distances, both EDOF lenses provided a significantly superior spectacle independence rate compared with the monofocal group (*p* ≤ 0.0001). Regarding overall patient satisfaction, at 6 months, 84% of the patients in the AT LARA 829MP group were completely or very satisfied, compared with 83.3% in the TECNIS Symfony group and 75.8% in the CT ASPHINA 409MP group (*p* ≥ 0.07).

### Safety

At 6 months, only one eye in the AT LARA 829MP group had a decentred IOL with a decentration of 0.5 mm. There was one case of slight tilt which had resolved at the final assessment.

At 6 months, mean IOP ranged from 13.34 ± 2.51 to 13.70 ± 2.46 mmHg across all groups.

The following adverse events were reported during the course of the follow-up (AT LARA 829MP; TECNIS Symfony; CT ASPHINA 409MP): 11 cases of punctate keratitis (3; 6; 2), 6 cases of cystoid macular oedema (1; 4; 1) and 4 cases of Nd:YAG capsulotomy (3; 0; 1). None of these events was considered as a serious adverse event. There were no cases of hypopyon, endophthalmitis, lens dislocation, pupillary block or retinal detachment.

## Discussion

The objectives of any cataract surgery are to achieve good visual acuity and to improve the quality of life for the patient. However, with recent developments in intraocular lenses, we now also strive to provide a sense of well-being and to enable the patients to carry out most of their daily activities such as driving; using computers, tablets and mobiles; and hobbies such as reading and sewing without glasses. The selection of the best IOL for a patient should not solely rely on the technical features of the lens. The patient demands, as well as his or her personal lifestyle and preferences (reading, driving, outdoor hobbies, etc.), should also be taken into consideration.

This study primarily investigated the depth of focus and also investigated other visual and safety parameters of two EDOF IOLs compared with one monofocal IOL control group as recommended by both the ANSI Z80.352018 and the American Academy of Ophthalmology (AAO) Task Force Consensus Statement for Extended Depth of Focus intraocular Lenses [11]. To our knowledge, this is the first study reporting comparative results for the AT LARA 829MP.

Looking at the one-sided depth of focus, there was a greater depth of vision with the AT LARA 829MP compared with the TECNIS Symfony at the three visual acuity thresholds evaluated. The effect was statistically significant under monocular vision but not under binocular conditions. At the benchmark threshold of 0.2 logMAR, the TECNIS Symfony IOL reached a defocus of − 0.7 D, while the AT LARA 829MP remained above this threshold up to a defocus of − 1.5 D. It is interesting to note that even the monofocal IOL outperformed the TECNIS Symfony at 0.2 logMAR. At the visual acuity level of 0.3 logMAR, the difference in depth of vision between the AT LARA 829MP and the TECNIS Symfony corresponded to approximately 13 cm. Correspondingly, between the TECNIS Symfony and the CT ASPHINA 409MP, the difference was 34 cm, and between the AT LARA 829MP and the CT ASPHINA 409MP, the difference was 47 cm. The monocular depth of focus in this study for the AT LARA 829MP ranged from 0.87 D at 0.1 logMAR to 1.75 D at 0.3 logMAR.

All three IOLs restored distance visual acuity producing excellent results with little difference between the IOLs. In particular, there was no reduction in CDVA with the EDOF lenses compared with the monofocal group, with the mean difference between the groups being less than one ETDRS line. The results for the AT LARA 829MP were very similar to those reported by Schallhorn et al. (2019) where mean binocular UDVA was − 0.05 ± 0.09 logMAR and mean binocular UNVA was 0.26 ± 0.14 logMAR [12].

Regarding intermediate and near visual acuity, there was a significant difference between the EDOF IOLs and the monofocal IOL for binocular and monocular vision at 6 months and both EDOF IOLs were comparable with regard to uncorrected near and intermediate visual acuities. Intermediate visual acuity is increasingly important to many patients to facilitate working at computers and for seeing car instruments. In this study, the AT LARA 829MP provided a level of visual acuity equal to or better than 0.3 logMAR from 52 cm and the TECNIS Symfony from 66 cm. In contrast, the CT ASPHINA 409MP only provided a good quality of vision from 100 cm. Reading newspapers typically requires a visual acuity of 0.4 logMAR at 40 cm, while a higher level of visual acuity is needed for fluid reading [13]. At 6 months, both EDOF IOLs had visual acuity well above 0.4 logMAR at intermediate distances. Regarding near vision, both EDOF IOLs would allow good functional vision, while the CT ASPHINA 409MP, as expected for a monofocal, would not support functional near vision (DCNVA: 0.50 logMAR).

Visual acuity outcomes were consistent with the results from the patient questionnaire with a majority of patients reporting no difficulties at all for activities requiring distance vision (e.g. driving, outdoor activities) and greater difficulties for activities requiring near vision (e.g. reading, sewing). Driving at night was reported in the three groups as being more difficult than driving at daytime. It is generally accepted that following cataract surgery very few patients experience difficulties with daytime driving. Another study by Mönestam et al. found that 43% of patients reported difficulties with night-driving due to glare, with the worst visual acuity being experienced in low-contrast conditions of less than 20/50 (0.4 logMAR) [14], which was consistent with our findings. It is accepted that reduced visual acuity to 20/40 can have a significant impact on overall night-time driving performance [15]: in our study, all IOLs exceeded this level on average. Patient questionnaire outcomes were also consistent with the contrast sensitivity results. Under photopic conditions, contrast sensitivity was comparable to the normal range for this population at all spatial frequencies [16]. But as expected, contrast sensitivity was reduced under mesopic and mesopic with glare conditions. Contrast sensitivities are reduced for the two EDOF lenses compared with those for the monofocal IOL, although statistical significance was only reached for spatial frequencies of 6 cpd or more.

Refractive predictability was good in the three groups with more than 95% of the eyes within ± 1.0 D of the target value. With multifocal lenses, any degree of residual refractive defect reduces the quality of vision at any distance compared with monofocal IOLs [17, 18]. The success of presbyopia correction is highly dependent on the degree of accuracy of post-operative refraction, and even small errors in the measurement of the eye dimensions will lead to refractive shifts that might cumulatively impact the quality of vision of the patient post-surgery. The management and treatment of the meibomian glands, the quality of the ocular surface or the level of precision of the biometers to measure the anterior and posterior corneal curvature are all important factors to be considered to determine the most suitable IOL power to be implanted. EDOF IOLs are expected to be more tolerant to moderate refractive shift and in that respect easier to use than other types of IOLs. Additional studies would however be required to further evaluate and confirm the tolerance to refractive errors of EDOF IOLs.

One limitation of this study comes from the use of the FrACT for the evaluation of visual acuity. The FrACT is an automated procedure for self-administered measurement of visual acuity. It uses optotypes including Landolt ring, tumbling E and Sloan letters. The FrACT has been validated and a lot of literature is available to support its use [19–21]. It offers in particular some advantages over conventional chart testing with respect to objectivity and reliability [19]. However, comparison with previously published literature might be limited as the FrACT computerised charts might yield lower results than conventional chart testing (ETDRS chart in particular) [22]. Although it does not compromise the internal validity of the study, comparison with previously published literature should be carried out with caution.

In conclusion, in our study, the AT LARA 829MP exceeded the AAO taskforce requirement for EDOF IOLs [11] (defined as an IOL with a monocular depth of focus at least 0.5 D wider than a monofocal control at 0.2 logMAR) with a difference of 0.62 D with the monofocal control, but the TECNIS Symfony failed to comply with the definition with a difference of only 0.22 D compared with the monofocal control. The study demonstrated good visual outcomes following implantation of both EDOF lenses. Vision at all distances was comparable between the two EDOF lenses and was better than the monofocal at intermediate and near distances. Photic phenomena, spectacle independence and patient satisfaction were comparable between the EDOF groups. These results confirm previous studies which found that EDOF IOLs produce good optical and visual outcomes at all distances, enabling the majority of patients to be spectacle-independent for most daily tasks.
